# Acupuncture combined with traditional Chinese medicine preparation for the treatment of marrow suppression after chemotherapy: A protocol for systematic review and meta-analysis

**DOI:** 10.1097/MD.0000000000027646

**Published:** 2021-10-29

**Authors:** Qiongjie Zhu, Wenjin Xu, Xuesong Li

**Affiliations:** Department of Traditional Chinese Medicine, Wuhan Asia General Hospital, Wuhan, Hubei, China.

**Keywords:** acupuncture, chemotherapy, marrow suppression, meta-analysis, protocol, traditional Chinese medicine preparation

## Abstract

**Background:**

From the perspective of evidence-based medicine, the efficacy and safety of combined therapy for marrow suppression after chemotherapy is still unclear. Given that there is no high-quality meta-analysis to incorporate existing evidence, the purpose of this protocol is to design a systematically review and meta-analysis of the level I evidence to ascertain the efficacy and safety of acupuncture combined with traditional Chinese medicine preparation for marrow suppression after chemotherapy.

**Methods:**

The following databases will be searched electronically by keyword combination mode: 4 British literature databases including PubMed, EMBASE, Scopus, and Cochrane Library, and 4 Chinese literature databases, including Chinese national knowledge infrastructure, VIP, and Wan fang database. The randomized controlled trials on acupuncture plus traditional Chinese medicine preparation for marrow suppression after chemotherapy will be included. The primary outcome is the elevation of hemoglobin, platelets, leukocytes, and neutrophils. The other outcomes include clinical symptoms, quality of life, and absolute value of reticulocyte. Risk bias analysis of the studies will be performed independently by 2 reviewers using the Cochrane Risk of Bias Assessment Tool.

**Results:**

The review will add to the existing literature by showing compelling evidence and improved guidance in clinic settings.

**Conclusion:**

This protocol will provide a reliable theoretical basis for the following research.

## 1 Introduction

Cancer encompasses a group of diseases in which cells harbor the ability to exhibit uncontrolled proliferation with the potential to invade and undergo metastasis to other parts of the body. Cancer is a chronic health condition with rapidly increasing morbidity and mortality in all regions of the world.^[1]^ In 2018, an estimated 18.1 million new cancer cases and 9.6 million deaths occurred worldwide.^[2]^ Lung cancer, breast cancer, prostate cancer, and colorectal cancer are the cancers that have the highest incidence, whereas lung, colorectal, stomach, and liver cancers result in the greatest number of deaths due to cancer. The health burden of cancer is increasing in China, with approximately 3.6 million new cancer cases and 2.2 million deaths each year. In summary, cancer is the leading cause of human death and is considered a serious threat to improving life expectancy in countries around the world.^[3]^

Radiotherapy and chemotherapy are the main treatments for cancer. However, the efficacy of chemotherapy has reached a bottleneck and may cause many side effects, especially bone marrow suppression. In China, traditional Chinese medicine (TCM) preparation combined with chemotherapy effect is remarkable. A number of studies have found that the combination of chemotherapy and TCM preparation can improve the sensitivity of chemotherapy and reduce the side effects of chemotherapy.^[4–6]^ A study of the TCM rikkunshito combined with chemotherapy found that the TCM preparation combined group had a higher 1-year survival rate.^[7]^ Many clinical studies have shown that TCM preparation can reduce the incidence of bone marrow suppression and gastrointestinal reactions in chemotherapy.^[8]^ In the East, acupuncture is an ancient non-pharmacological treatment for chemotherapy-induced bone marrow suppression with relatively low cost and fewer side effects. In addition, increasing clinical studies have shown that acupuncture has a good therapeutic effect on marrow suppression caused by chemotherapy.^[9–11]^

Based on these grounds, we have confused an important clinical question: Is acupuncture combined with TCM more effective and safer than monotherapy for patients with marrow suppression after chemotherapy? Unfortunately, from the perspective of evidence-based medicine, the efficacy and safety of combined therapy for marrow suppression after chemotherapy is still unclear. Given that there is no high-quality meta-analysis to incorporate existing evidence, the purpose of this protocol is to design a systematically review and meta-analysis of the level I evidence to ascertain the efficacy and safety of acupuncture combined with TCM preparation for marrow suppression after chemotherapy.

## 2 Materials and methods

### 2.1 Search strategy

The following Chinese and English databases will be searched electronically by keyword combination mode: 4 British literature databases including PubMed, EMBASE, Scopus, and Cochrane Library, and 4 Chinese literature databases, including Chinese national knowledge infrastructure, VIP, and Wan fang database. We also limit the search time to December 2021 on the treatment of marrow suppression after chemotherapy with TCM plus acupuncture. The search strategy of other electronic databases is similar to that of PubMed. The search terms in Chinese databases are the translation of the above words. Two searchers will independently draft and carry out the search strategy, and the third member will further complete it. References within included articles are reviewed to include articles that are not included within our literature search. The systematic review protocol has been registered on Open Science Framework registries. The registration number is 10.17605/OSF.IO/DRP5K. Since this study is on the basis of published or registered studies, ethical approval and informed consent of patients are not required.

### 2.2 Inclusion and exclusion criteria

Included studies are considered eligible if they met the Population, Intervention, Comparator, Outcomes, and Study design criteria as follows:

Population: cancer patients treated with chemotherapy;
Intervention: group with acupuncture combined with TCM preparation;
Comparator: group with acupuncture alone or TCM preparation alone;
Outcomes: the primary outcome is the elevation of hemoglobin, platelets, leukocytes, and neutrophils. The other outcomes include clinical symptoms, quality of life, and absolute value of reticulocyte.
Study design: randomized controlled trials.

Exclusion criteria include observational studies, non-randomized controlled trials, review articles, studies with a sample size <30, and studies with insufficient outcome data.

### 2.3 Data extraction

Two reviewers will independently extract data from selected studies and fill in the data extraction form which has already been developed. The authors of trials will be contacted for further details if necessary.

We will extract the following information:

1. General information for the articles including the first author, year, country, sample size
2. Demographic characteristics including gender, age and other information including tumor type and tumor stage
3. Intervention parameters including type of acupuncture, acupoints used, chemotherapy drugs, type of TCM preparation treatment, and frequency and duration of treatment
4. Outcome information including results, adverse events, costs and quality of life.

### 2.4 Data analysis

RevMan V.5.4 (Copenhagen, The Nordic Cochrane Centre, The Cochrane Collaboration 2020) will be employed for data analysis when meta-analysis is possible. The mean difference (MD) with 95% confidence intervals (CIs) will be used to assess continuous outcomes, while the risk ratio (RR) with 95% CIs will be used for dichotomous data. Heterogeneity will be assessed by visually inspecting the forest plot and investigated by χ^2^ (significance level: *P* < .10) and I^2^ statistics. I^2^ <50% will be taken as evidence of no statistical heterogeneity, while I^2^ ≥50% will be considered to indicate substantial heterogeneity. The causes of heterogeneity among study results will be explored through subgroup analysis or sensitivity analysis. If I^2^ <50%, the RR and MD will be calculated by a fixed-effects model. If I^2^ ≥50%, a random-effects model, will be used to synthesize the data and subgroup analysis or sensitivity analysis will be conducted to explore the causes of heterogeneity including clinical or methodological reasons. We may conduct narrative synthesis if meta-analysis is not appropriate (e.g., incidence of adverse events of acupuncture). We will generate funnel plots to detect publication bias or small-study effects using sufficient numbers of included studies (at least 10 studies).

### 2.5 Risk of bias

Risk bias analysis of the studies will be performed independently by 2 reviewers using the Cochrane Risk of Bias Assessment Tool for the following criteria: random sequence generation, allocation concealment, blinding of outcome assessment, incomplete outcome data, selective reporting, and other bias. When reviewers have different opinions, consensus is reached through discussion. Evidence from research studies is ranked as having either “high,” “low,” or “unclear” risk of bias.

## 3 Discussion

The main cause of marrow suppression is that chemotherapy cannot only attack tumor cells, but also inhibits the strong proliferation and low differentiation of bone marrow cells, inhibits all immature cells with proliferative function, and eventually leads to bone marrow suppression.^[12]^ At present, the treatment options for chemotherapy-induced marrow suppression are mainly drug intervention, with high cost and unsatisfactory efficacy. Therefore, the management of chemotherapy-induced myelosuppression is a challenge for patients, physicians, and government health authorities.^[13]^ Therefore, there is an urgent need to seek a non-drug intervention to treat myelosuppression induced by chemotherapy. Unfortunately, from the perspective of evidence-based medicine, the efficacy and safety of combined therapy for marrow suppression after chemotherapy is still unclear. Given that there is no high-quality meta-analysis to incorporate existing evidence, the purpose of this protocol is to design a systematically review and meta-analysis of the level I evidence to ascertain the efficacy and safety of acupuncture combined with TCM preparation for marrow suppression after chemotherapy.

## Author contributions

**Conceptualization:** Qiongjie Zhu, Wenjin Xu.

**Data curation:** Qiongjie Zhu, Wenjin Xu.

**Funding acquisition:** Xuesong Li.

**Investigation:** Qiongjie Zhu, Wenjin Xu.

**Methodology:** Xuesong Li.

**Project administration:** Xuesong Li.

**Software:** Qiongjie Zhu, Wenjin Xu.

**Supervision:** Xuesong Li.

**Validation:** Wenjin Xu.

**Visualization:** Wenjin Xu.

**Writing – original draft:** Qiongjie Zhu, Wenjin Xu.

**Writing – review & editing:** Xuesong Li.
